# Evoking Context with Contrastive Stress: Effects on Pragmatic Enrichment

**DOI:** 10.3389/fpsyg.2015.01779

**Published:** 2015-11-26

**Authors:** Chris Cummins, Hannah Rohde

**Affiliations:** Linguistics and English Language, University of EdinburghEdinburgh, UK

**Keywords:** question under discussion (QUD), scalar implicature, presupposition projection, coreference, focus placement

## Abstract

Although it is widely acknowledged that context influences a variety of pragmatic phenomena, it is not clear how best to articulate this notion of context and thereby explain the nature of its influence. In this paper, we target contextual alternatives that are evoked via focus placement and test how the same contextual manipulation can influence three different phenomena that involve pragmatic enrichment: scalar implicature, presupposition, and coreference. We argue that focus placement influences these three phenomena indirectly by providing the listener with information about the likely question under discussion (QUD) that a particular utterance answers (Roberts, 1996/2012). In three listening experiments, we find that the predicted interpretations are indeed made more available when focus placement is added to the final element (to the scalar adjective, to an entity embedded under the negated presupposition trigger, and to the predicate of a pronoun). These findings bring together several distinct strands of work on the effect of focus placement on interpretation all in the domain of pragmatic enrichment. Together they advance our empirical understanding of the relation between focus placement and QUD and highlight commonalities between implicature, presupposition, and coreference.

## Introduction

The study of pragmatics examines how hearers infer meaning beyond that which is explicitly expressed by the speaker. This process crucially depends upon the consideration of what is not said as well as what is said. To take one much-discussed example, quantity implicature has traditionally been assumed to rely on the hearer's ability to identify and reason about more informative alternatives that the speaker could have uttered. For example, a hearer is expected to reason that a speaker who utters (1) had available to them a stronger statement, as in (2), and that because the speaker chose not to utter (2), the hearer is entitled to infer the classic scalar implicature from (1), namely the negation of (2).

1. Mary saw some of John's children today.
2. Mary saw all of John's children today.

A major concern arising from this line of reasoning is what we mean by alternatives that the speaker “could have uttered.” As Grice (1975) sketched out, we expect that cooperative speakers will adhere to several principles of interaction. They will not make statements which are false or for which they lack evidence, they will produce utterances that are relevant, they will be concise, and they will make their contribution only as informative as is required for the current purposes of the dialogue in which they are engaged.

The net effect of this is to substantially narrow down the space of alternatives that are pragmatically consequential in a particular set of circumstances. For instance, implicatures are predicted not to be available based on informationally stronger statements about which the speaker is not knowledgeable, because the speaker could not have made these statements without violating Grice's quality maxim—therefore, the speaker's unwillingness to utter them is not intended to signal their falsity.^1^ Similarly, implicatures should not be available when the additional information provided by the stronger statement would have been irrelevant to the current discourse purpose, putatively because the speaker could not convey this additional information without violating the maxim of relation. These predictions have been borne out experimentally (Breheny et al., 2006; Goodman and Stuhlmüller, 2013).

Nevertheless, as Grice himself acknowledged, the issue of determining whether or not a potential utterance would have been relevant to the current discourse purpose, had it been uttered, is not a straightforward matter. Roberts (1996/2012) approaches this by appeal to the notion of Question Under Discussion (QUD), which she defines as the immediate topic of discussion and which she takes to proffer a set of relevant alternatives. A felicitous assertion is, on this view, one which bears upon the QUD by choosing among the alternatives that it proffers. For instance, a QUD of “How many of John's children did Mary see today?” would proffer a set of alternatives including “some of them” and “all of them,” and both (1) and (2) would be felicitous responses to this QUD. If that particular question is indeed the one under discussion, the hearer of (1) is expected to identify that (2) would have been a felicitous alternative and (given some additional assumptions) understand (1) to implicate the negation of (2).

What if the QUD is not explicitly given, though? Roberts (1996/2012) takes the view that the QUD is often merely implicit and has to be inferred on the basis of other considerations. Specifically, she cites the use of prosodic focus as a cue to QUD in English. As she puts it (2012: 27), “assertions, like questions, are conventionally associated with a set of alternatives, although these alternatives are presupposed by the prosody rather than proferred” (see also Büring, 2003). This proposal develops the observation of Jackendoff (1972) that the prosody of an assertion constrains the set of questions to which it could be an answer: on Roberts's account, we can go further and use the prosody of an assertion to identify relevant alternatives that could have been uttered in place of the actual assertion.

In this paper, we discuss the use of focus marking to evoke sets of alternatives and experimentally test the impact of such alternatives on three distinct pragmatic phenomena: scalar implicature, presupposition cancelation, and coreference. We argue that a QUD-based analysis potentially offers a unified explanation of what appear, on the surface, to be very different pragmatic consequences; and we introduce novel experimental data to show that these effects are indeed evident in comprehenders' behavior.

## Some pragmatic consequences of focus manipulation

### Scalar implicature

Since Horn (1972), scalar implicatures have been widely discussed as a special case of quantity implicature. As Geurts (2010, p. 49) puts it, “the distinctive feature of scalar implicatures is that we can use lexical substitution to generate the relevant alternatives from the sentence uttered.” This is evident in the case of (1) above: the alternative, (2), is generated simply by replacing the informationally weaker “some” with the stronger “all.” We can think of < *some, all*> as constituting an informational scale.

A widespread intuition within the literature is that (at least some) scales of this form are privileged in terms of their pragmatics, in that the use of a weak term from one of those scales robustly tends to implicate the falsity of the corresponding utterance with any stronger scalemate. Indeed, for cases such as < *some, all*>, the inference (that “some” tends to mean “not all”) is sufficiently robust to have motivated accounts in which it is generated by default (Levinson, 2000) or is grammaticalised (Chierchia et al., 2012). From a QUD point of view, we can understand this observation as a generalization about the kinds of question to which a weak scalar is an appropriate answer: that is, whenever a weak scalar is a felicitous answer to a given QUD, any stronger scalemate would likewise be a felicitous answer. Consequently, it is generally appropriate for the hearer to embark on pragmatic reasoning concerning the stronger alternative (thus deriving the implicature), safe in the knowledge that the stronger alternative would indeed have been an appropriate thing for the speaker to have uttered, had the speaker known it to be true.

The extensive recent experimental literature on scalar implicature has demonstrated that things are not quite so clear-cut as had previously been supposed. In fact, there is considerable variability between participants as to whether or not they endorse scalar inferences such as “some” -> “not all,” with the overall response rates also depending on task factors (see Katsos and Bishop, 2011 for a review). A possible explanation for this is that the tendency of a particular weak scalar to evoke a suitable context for implicature (i.e., a context in which the stronger alternative would also have been felicitous) is not necessarily as strong as had been postulated. This may reflect the fact that, under certain circumstances, it is possible to use weak scalars in contexts in which their stronger scalemates are not judged to be especially relevant, as was shown by Breheny et al. (2006). For instance, in the context of (3), the use of the weak scalar “or” [as in (4)] already adequately answers the question of whether there is at least one person who will be available. The use of the stronger scalemate “and” [as in (5)] would not necessarily be warranted, inasmuch as the extra information it conveys is not essential for the current discourse purpose. Indeed, (5) could be altogether less useful than (4), to the extent that it introduces an ambiguity concerning whether Kate and Rob will be available separately. Correspondingly, readers/hearers do not tend to infer, on the basis of (4), that (5) is false.

(3) Can anyone cover for me next week?
(4) Kate or Rob will be available.
(5) Kate and Rob will be available.

Given that scalar implicatures are not obligatory in all contexts, we can ask whether their availability is sensitive to the kind of focus manipulation discussed by Roberts (1996/2012). The intuition is that placing focus on the weak scalar term emphasizes its potential for being substituted: that is, that the relevant alternatives to the utterance involve the substitution of some other lexical item in place of the weak scalar. For instance, by stressing “some,” we call particular attention to the possibility that other items such as “all” could be used in its stead, and consequently feed these into the calculation of potential pragmatic enrichments. By hypothesis, the use of a weak scalar already tends to evoke this set of alternatives, but [as shown by cases such as (4)] this is not invariably the case. We might therefore expect that placing focus on the weak scalar will increase the rate at which comprehenders infer scalar implicatures.

This hypothesis has been partially tested by placing utterances containing elements from the < *or, and*> scale (Zondervan, 2006) and the < *some, all*> scale in contexts in which a preceding question designates the target utterance's intended focus structure (Zondervan et al., 2009). Using dialogue fragments like (6) and (7), Zondervan et al. show that comprehenders draw significantly more implicatures (agreeing more often with the statement that “not all pizzas were delivered”) in the context of a question that evokes the stronger scalar alternative (6) than one that does not (7).

(6) A: Were all pizzas delivered?
      B: Some pizzas were delivered.
(7) A: Were some pizzas delivered?
      B: Some pizzas were delivered.

Intuitively, B's utterance in (6) has focus on “some,” and would perhaps most naturally be read aloud with focal stress on that word, whereas B's utterance in (7) does not, and would be read with stress on “were.” The finding therefore coheres with Roberts' account. However, it should be noted that (6) does not merely evoke the stronger alternative “all” through the presumed focus placement in B's utterance, but explicitly introduces it in A's utterance. By contrast, (7) makes no mention of “all.” Moreover, in (7), B's utterance is unnecessarily verbose (B could just reply “yes”), and it seems possible that a reader could doubt B's full cooperativity. Therefore, it might be premature to attribute the difference in judgments between (6) and (7) entirely to focus considerations.

Recent experimental work points to a role for the combination of preceding context and explicit manipulations of prosody in the interpretation of scalar implicatures. De Marneffe and Tonhauser (2015) test for effects of prosody in two different contexts which provide the background against which to interpret a scalar adjective—either an explicit utterance similar to A's polar question in (7) or a preceding statement regarding speaker A's commitments. In both contexts, a rise-fall-rise intonation on B's subsequent utterance containing the scalar adjective leads listeners to report stronger degrees of belief in the pragmatically strengthened meaning compared with a neutral intonation. However, this leaves open the question of whether prosody alone can shift the hearer's understanding of what the preceding context is likely to contain, in such a way as to influence the pragmatic interpretation of the utterance. Under a QUD-based account, this should be possible: an utterance's prosody is one of the cues that listeners use to infer what question the utterance may be a relevant answer to.

As in the case of much of the experimental research on scalar implicature, the existing work on focus effects has attended to a limited number of potential scales. More recent work by Van Tiel et al. (2014) demonstrates substantial variability among potential implicature scales with respect to the availability of their corresponding implicatures. They demonstrate that, within a neutral context, the rates of endorsement of 43 candidate scalar implicatures ranged from 4% (e.g., “tired” +> “not exhausted”) to 100% (e.g., “sometimes” +> “not always”), with “some” +> “not all” very near the top of the range at 96%. This variability raises the question of whether the effect of focus in promoting scalar implicature is general across a broad range of triggers. On the one hand, “some” and “or” (which was not tested by Van Tiel et al.) may be atypically strong implicature triggers, and consequently the effect of focus may be particularly clear-cut in these cases, as the stronger scalar alternatives are especially susceptible to being evoked. On the other hand, it is possible that “some” and “or” could be influenced less by the presence of focus, as they already evoke the stronger scalar alternatives to the fullest extent possible even without additional stress being introduced.

Experiment 1 of this paper evaluates the availability of a variety of different scalar implicatures, using intonation to signal focus placement on a weak scalar. The study goes beyond prior work that has manipulated the preceding context against which a scalar is interpreted (Zondervan et al., 2009; de Marneffe and Tonhauser, 2015). If hearers can instead make use of focus placement on an utterance in isolation to recover a likely QUD that is operative in the context, that QUD and the set of alternatives it evokes is predicted to influence the perceived availability of the scalar implicature.

As we will show, this prediction is borne out. However, the finding follows from the fact that scalar implicatures necessarily depend on the presence of alternatives (“scalemates”). Arguably a more substantive result would be a demonstration that the manipulation of focus influences scale-independent pragmatic phenomena. To that end, we next consider presupposition and coreference.

### Presupposition cancellation

The tendency of content to project from under the scope of negation has long been identified as diagnostic of presupposition, as opposed to other forms of non-asserted content. For instance, both (8) and its negation (9) presuppose (10). By appeal to accommodation, either (8) or (9) can be used to convey the fact of (10) to a hearer who was not previously aware of it.

(8) John quit smoking.
(9) John didn't quit smoking.
(10) John smoked, at some point prior to the time of utterance.

Nevertheless, it is quite possible for a presupposition under the scope of negation to be canceled, or to fail to project to the discourse level. (11) is an apparently felicitous example.

(11) John didn't quit smoking—he never smoked in the first place.

In principle, the acceptability of (11) suggests that the hearer is confronted with a difficult problem when she encounters an utterance like (9)—should the presupposition (10) be added to her discourse model, even though this might turn out to be an erroneous inference? Or should she wait until it is made clear whether or not the speaker intends to communicate (10)? This puzzle appears to vitiate the communicative benefits of being able to exploit accommodation to convey a presupposition.

It may be possible to solve this puzzle by appeal to the notion of QUD. An utterance like (11), in which a presupposition is apparently triggered (in this case, by the use of “quit”) and then canceled, may suggest the presence of a current QUD that already assumes that presupposition. For (11), the QUD appears to be something like “Did John quit smoking?” The set of proffered answers then effectively comprises (8) and (9), both of which contain the presupposition trigger “quit,” and the speaker's subsequent utterance of one of them does not constitute an attempt to convey the presupposition. If the hearers are aware of, or can infer, the existence of such a QUD, then they should not take the speaker's utterance of “quit” as necessarily committing the speaker to the belief that John used to smoke. By contrast, an utterance like (9) is potentially compatible with a wider range of QUDs (for example, “What did John do after he saw his doctor?”), some of which proffer alternatives that do not involve the presupposition trigger “quit.” The subsequent use of “quit” thus represents the outcome of a choice on the part of the speaker, and consequently has the potential to convey meaning (i.e., the presupposition).

How might focus effects come into play here? A speaker who utters (9) neutrally, or placing stress on “didn't,” seems merely to evoke an alternative such as (8). This is compatible with a situation in which the QUD is “Did John quit smoking?” and the speaker does not wish to challenge the presupposition. However, a speaker who utters (9) but places focal stress on “John” appears to give rise to a different set of alternatives, involving all the people who might have quit smoking. This kind of focus appears to suggest a continuation such as (11) or (12).

(12) JOHN didn't quit smoking—you're thinking of Bill.

As far as the QUD is concerned, focus on “John” suggests that it is likely to be of the form “Who (didn't) quit smoking?” There is still a presupposition built into this question, namely that someone (in the universe of discourse) used to smoke at some point prior to the time of utterance, but the specific presupposition that John used to smoke is now absent.

The story is similar if stress is placed on “smoking.” In this case, the implied QUD is “What did John quit doing?” and the alternatives are the things that John might have quit (e.g., “drinking”). Again, the QUD encompasses a presupposition that John used to do something (of interest to the discourse purpose), but not specifically that he used to smoke.

If this line of reasoning is correct, then the hearer's inference from (9) to (10)—the projection of the presupposition from under the scope of negation to the discourse level—relies upon the assumption that (9) answers a QUD that presupposes (10). This inference will therefore be obstructed if focus is placed on “John” or “smoking.” In either case, the hearer will be encouraged to infer a QUD which does not presuppose (10), and hence not project the presupposition. This observation and variants of the QUD-based analysis have been outlined in similar form in several recent papers (Beaver and Clark, 2008; Cummins, 2014; Simons et al., to appear). Experiment 2 tests these predictions experimentally. As in our first experiment, Experiment 2 manipulates focus placement to influence the QUD a hearer infers, and as we show, this manipulation in turn modulates the projection of the presupposition from under negation.

We now turn to a phenomenon that is known to be sensitive to QUD but that has not been typically analyzed alongside implicature or presupposition: pronoun interpretation.

### Coreference

Assigning reference to pronouns gives rise to ambiguity in cases such as (15), where more than one suitable potential referent is present in the preceding context.

(15) Mary scolded Sue. She praised Bob.

An extensive literature posits a number of factors that comprehenders bring to bear on the process of pronoun interpretation. Some factors are taken to reflect surface structure—e.g., a preference for antecedents in subject position or a preference for grammatical role parallelism (Sheldon, 1974; Smyth, 1994; Stevenson et al., 1994). Other factors reflect deeper properties of the utterance such as the lexical semantics of the verb or its thematic role assignments (Caramazza et al., 1977; Stevenson et al., 1994; Arnold, 2001). An alternative approach (Hobbs, 1979; Kehler, 2002; Kehler et al., 2008) argues that such preferences emerge as a by-product of reasoning about the most likely interpretation of an utterance in relation to adjacent utterances. These intersentential relationships can be understood either as coherence relations or as QUDs which can influence pronoun interpretation (Rohde, 2008; Kehler and Rohde, under review).

In many discourse contexts, all of these approaches make the same prediction regarding a pronoun's preferred interpretation. However, an example like (15) reveals key differences and allows us to highlight the role of the inferred QUD. While parallelism and subjecthood preferences both favor the interpretation of “she” in (15) as referring to Mary, the status of the verb “scold” as a member of the class of so-called NP2-biased Implicit Causality (IC) verbs is posited to yield a preference for Sue, the referent filling the patient thematic role and appearing in object position (Garvey and Caramazza, 1974; Brown and Fish, 1983; Au, 1986; McKoon et al., 1993; Koornneef and van Berkum, 2006). This difference is unsurprising if the preferred interpretation of the pronoun is understood to depend on the coherence relation that is inferred to hold between the two sentences (Kehler et al., 2008).

If the second sentence in (15) serves as an explanation of the first, then the combination of the lexical semantics of “scold” and the causal coherence relation yields a preference to interpret “she” as the causally implicated referent of a scolding event, namely the scoldee, Sue (i.e., Mary scolded Sue because she_Sue_ praised Bob). In this case, we are led to assume some set of circumstances under which, from Mary's point of view, the action of praising Bob is worthy of reproach. This is taken to be a more plausible state of affairs than a reading in which Mary praising Bob (she_Mary_ praised Bob) stands as an explanation for Mary scolding Sue (although we might be able to imagine contexts in which this is conceivable). If instead the second sentence is interpreted to be relevant to the first via a discourse relation centered on parallelism, then what is important is the similarity of the entities and actions in the two sentences, e.g., Mary as the Agent of the scolding event (and the subject of the first sentence) can be mapped to Mary as the Agent of the praising event (and the subject of the second sentence), with Sue and Bob as the respective Patients. The fact that “scold” and “praise” are both members of the class of agent-patient IC verbs while differing in affect supports the inference of a contrast relation (i.e., Mary scolded Sue, but she_Mary_ praised Bob).

The different interpretations of (15) seem to suggest the existence of different QUDs. Under the parallel interpretation, both sentences can naturally be construed as partial answers to a single QUD “What did Mary do?” Under the causal interpretation of (15), the sentences, respectively, answer two distinct QUDs to the effect of “What did Mary do?” and “Why did she do that?”

On this analysis, we would again predict that the interpretative preference for the pronoun would be influenced by the presence of focal stress in the second sentence. Suppose that stress is placed on the word “Bob” in (15). For the same reasons discussed earlier, this suggests that the QUD in effect at the second sentence is “Who did X praise?,” where X denotes the referent of “she,” i.e., Mary or Sue. If X = “Mary,” then the question that the second sentence partially answers is “Who did Mary praise?,” which is a subquestion of “What did Mary do?,” which in turn is the QUD most likely to be operable for the first sentence. By contrast, if X = “Sue,” the second sentence partially answers “Who did Sue praise?,” which is not a subquestion of “What did Mary do?” Moreover, it is not transparently a subquestion of “Why did Mary scold Sue?,” although it could be interpreted as such under some additional assumptions. It is not, after all, a likely state of affairs that Mary scolding Sue was caused by Sue praising anyone (though the fact that one may attempt to formulate such a scenario is a testament to the bias in favor of causal coherence relations following IC verbs). A similar argument applies if focal stress is placed on “praised”: again, if “she” refers to Mary, the second sentence is a partial answer to the first sentence's likely QUD, whereas if “she” refers to Sue it is not.

In summary, then, on QUD grounds, we would expect the placement of stress on “praised” or “Bob” in the second sentence of (15) to promote the parallel interpretation, in which the pronoun refers to the subject, Mary, over a causal interpretation, in which the pronoun refers to the object, Sue. A similar theoretical case is made by Kehler (2005) for differences in the interpretation of an ambiguous pronoun depending on the coherence relation that is inferred to hold between two adjacent clauses, although in Kehler's example [see (16)], subject coreference is favored by the causal coherence relation and object coreference by parallelism.

(16) Powell defied Cheney, and Bush punished him.

Kehler argues that the parallel interpretation is associated with accent placement on each word of the second clause, whereas the causal interpretation leaves the final word unaccented. Focus marking is thus predicted to influence the inferred relation or question under discussion. Experiment 3 uses IC contexts to test the prediction that accent placement can guide listeners' inferred relation, which in turn has repercussions for coreference. We replicate the widely reported NP2 bias (for pronoun coreference with the object of NP2-biased verbs) and present the first experimental evidence of this novel effect showing that IC biases are reduced when there is focus placement on the predicate of the subsequent clause.

### Interim summary

We have argued in the preceding subsections that the same form of manipulation—introducing focal stress on a particular constituent—should have pragmatic consequences of an apparently diverse nature across a range of structures. In the case of scalar implicatures, we argue that focusing a weak scalar term should increase the availability of the implicature, although the effect of this may vary between scales. In the case of presupposition, we argue that focusing any of various arguments of a presupposition trigger may result in the presupposition being less likely to project from under the scope of negation. And in the case of pronominal coreference, we argue that focusing the predicate of a subject-pronominal sentence is likely to promote a parallel interpretation of the pronoun over alternative causal readings. All of these consequences flow naturally from a view in which focus presupposes a set of alternatives, as argued by Roberts (1996/2012). The following sections present a short series of experimental studies designed to test these predictions.

Before we proceed, it is worth asking why these three phenomena have not previously been linked together via QUD. This may reflect a difference in emphasis over the fields' histories and their treatment of literal and inferred meaning. On the one hand, work on implicature has assumed that the literal message is easy to identify and that complexity emerges in the subsequent calculation of what is meant beyond that literal meaning. Likewise, in the case of presupposition and presupposition accommodation, the emphasis has been placed on identifying what additional meaning is at stake given the words used to convey a particular literal message. On the other hand, coreference models typically target the ambiguity in the literal message, specifically concerning which individual in the available set of entities in the preceding context is most likely to be referenced here. This in turn depends on inferences about the operative coherence relation. Only recently have these three areas been analyzed in terms of QUD. Pronouns historically were modeled primarily in terms of entity salience (see Ariel, 1990; Gundel et al., 1993; Arnold, 2001) and more rarely in terms of QUDs or coherence relations (Winograd, 1972; Hobbs, 1979; Kehler, 2002). Work in implicature and presuppositions has only recently focused on the importance of QUD (Breheny et al., 2006; Beaver and Clark, 2008; Zondervan et al., 2009; Cummins, 2014; de Marneffe and Tonhauser, 2015; Simons et al., to appear). Our studies represent the inevitable convergence of these separate research strands.

## Experiment 1: scalar implicature

This experiment uses a rating task to test the hypothesis that the availability of scalar implicatures is sensitive to QUD, as evoked via focus placement. Participants listen to sentences containing weak scalars in two conditions (neutral vs. focus) and then answer a question about the status of a stronger statement. The design is a within-participants and within-items manipulation.

### Participants

Seventy-seven English-speaking participants were recruited from Amazon Mechanical Turk, location restricted to the United States. After eliminating data from 12 bilinguals and 5 participants who failed to complete the task, data from 60 monolingual participants remained for the main analysis. Participants were paid between $1.80 and $2.50.

For this and the subsequent experiments, each participant was provided in advance with information about the procedure and gave informed consent. The experiments were conducted in accordance with the University of Edinburgh's ethics policy and the UKRIO Code of Practice for Research, and under the oversight of the departmental Ethics committee.

### Materials

Target stimuli consisted of 20 recorded sentences, each containing a weak scalar in sentence-final position, as in (17), interleaved with 20 sentences for Experiment 2. The full stimuli set is listed in Appendix A.^2^

(17) The view from the hotel window is pretty.

The target sentences were recorded in two conditions: neutral intonation and focus placement on the scalar. The stimuli were recorded by a native speaker of English (the first author of this paper). Note that any variability in the recordings of these two conditions would serve only to reduce our ability to observe a difference between conditions.

The experiment consisted of 40 items: The 20 target items for Experiment 1 were intermixed with 20 items for Experiment 2, which were likewise one-sentence items with variable intonation.

### Procedure

Participants accessed the experiment via a website linked within Mechanical Turk. Each participant listened to all 20 sentences, half in the neutral intonation condition and half in the focus placement condition. Across participants, each sentence appeared in both conditions. Participants were asked to listen to the sentence and answer a question about the speaker's intended meaning on a scale of 1 to 7. The text showing the question was visible on the screen during and after playback of the recorded sentence. Participants could replay the sentence as many times as they wished. Each item appeared on a page by itself, with a radio-button interface for participants to record their rating.

For the Experiment 1 target items, the question asked about a relevant stronger scalemate: For example, the question for the recording of (17) was (18), with answer “1” labeled as “unlikely” and “7” labeled as “likely.

(18) How likely is it that the view is not gorgeous?

The task took roughly 20 min.

### Results

**Figure 1:**
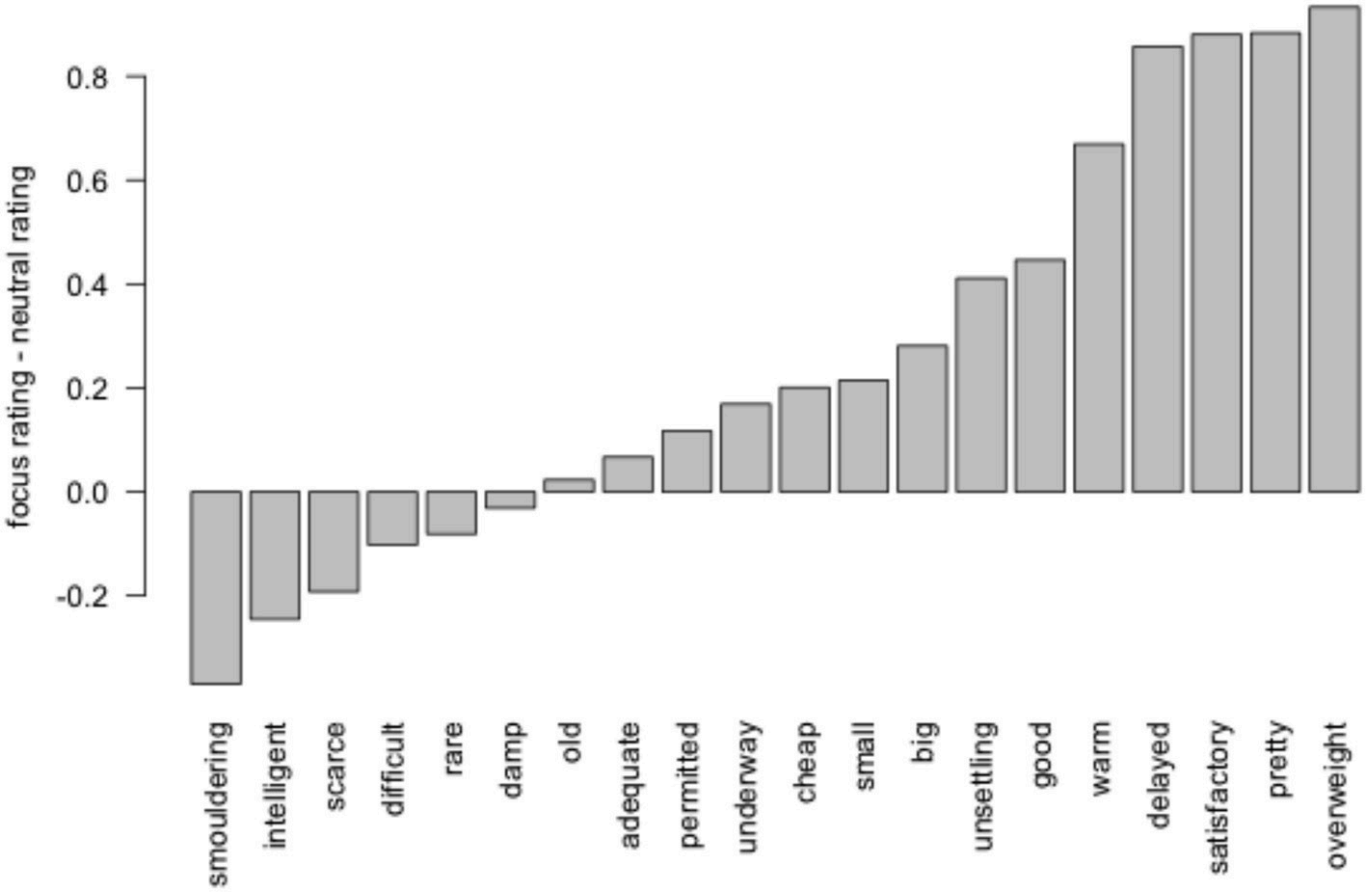
**Endorsement of pragmatic meaning for weak scalars in Experiment 1, by item**.

We modeled the ratings using a mixed-effect linear regression with a fixed effect of condition. All models reported in this paper contain random participant-specific and item-specific intercepts and slopes where permitted by the data (Barr et al., 2013). As predicted, participants endorsed the stronger statement (“not gorgeous”) more in the focus condition (mean = 5.05) than the neutral condition (mean = 4.74), showing a main effect of condition (β = 0.26, *t* = 2.707). We conducted a likelihood-ratio test between mixed-effects models differing only in the presence or absence of the fixed main effect of condition. The model comparison showed a main effect of condition (*p* < 0.05, 1 d.f.). Figure 1 shows the difference between ratings in the focus placement and neutral conditions, broken down by item.^3^

## Experiment 2: presupposition

This experiment uses a rating task to test the hypothesis that the projection of a presupposition under negation is sensitive to QUD, again evoked via focus placement. Participants listen to sentences containing presupposition triggers in two conditions (neutral vs. focus) and then answer a question about the status of the presupposition.

### Participants

Because the Experiment 1 and Experiment 2 stimuli were interleaved in a single task, the same participants from Experiment 1 also completed this experiment.

### Materials

Target stimuli consisted of 20 recorded sentences, each containing a presupposition trigger, as in (19), in either a neutral or focus condition. The focus condition placed a pitch accent on the last word of the sentence. This word was either part of an embedded clause under a factive trigger (e.g., *be sorry that the jewels were in the SAFE*) or was otherwise within the scope of the trigger by being mentioned as part of an argument of a trigger verb (e.g., *return to a job at CHRYSLER*) or as an adjunct (e.g., *finish a degree at HARVARD*).

(19) Bill doesn't regret arguing with his boss.

### Procedure

The procedure is described in Experiment 1 above. For the Experiment 2 target items, the question asked directly about the presupposition: For example, the question for the recording of (19) was (20), with answer “1” labeled as “unlikely” and “7” labeled as “likely.

(20) How likely is it that Bill argued with his boss?

### Results

**Figure 2:**
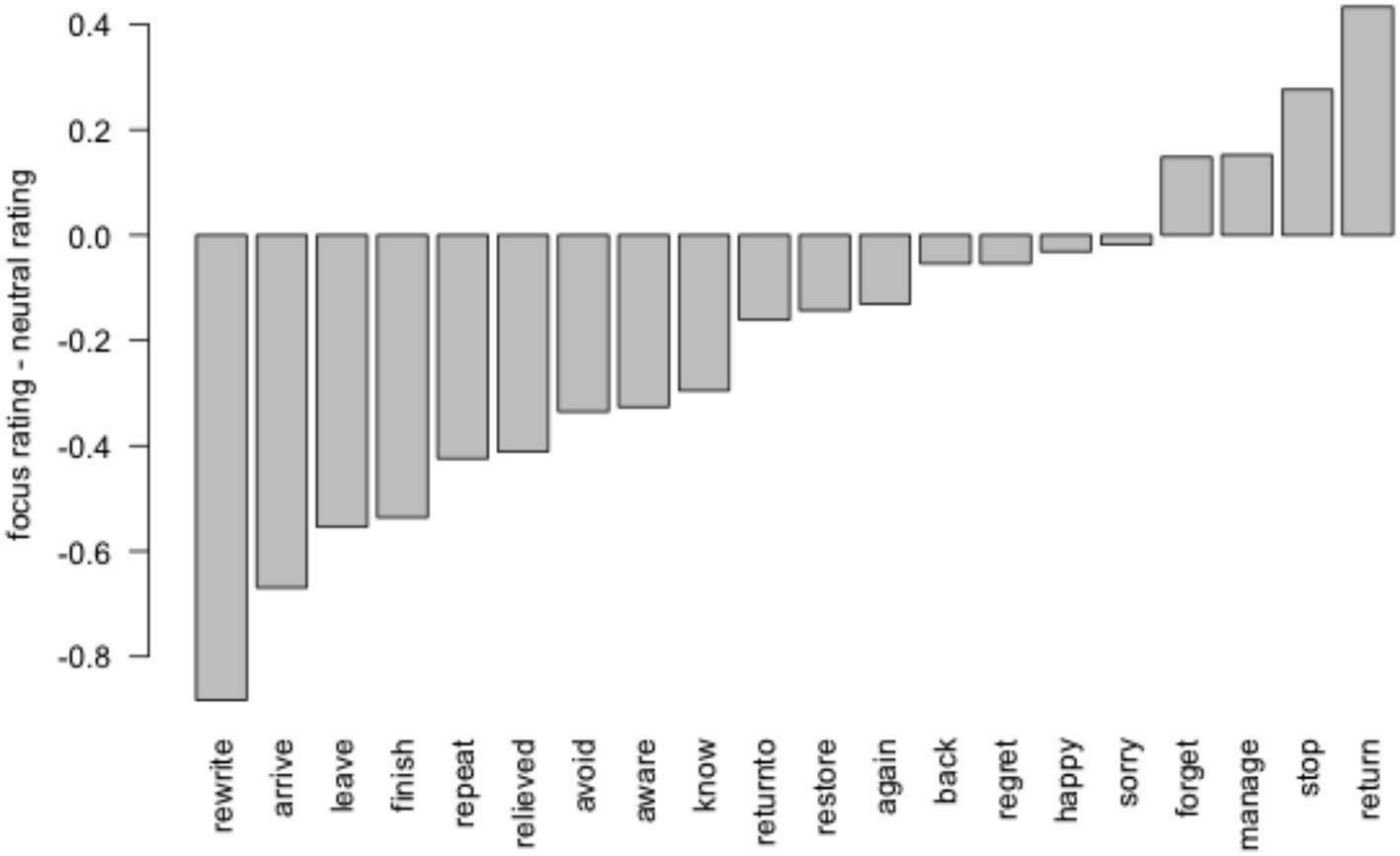
**Endorsement of the presupposed proposition in Experiment 2, by item**.

As predicted, participants gave lower ratings to the presupposed statement (“Bill argued with his boss”) in the focus condition (mean = 5.97) than the neutral condition (mean = 6.15). As in Experiment 1, we modeled the ratings using a mixed-effect linear regression with a fixed effect of condition. The effect of condition (β = 0.20, *t* = 2.30) was significant under model comparison (*p* < 0.05, 1 d.f.). Figure 2 shows the difference between ratings in the focus placement and neutral conditions, broken down by item.

## Experiment 3: coreference

This experiment uses a pronoun interpretation task to test the hypothesis that coreference is sensitive to QUD, again evoked via focus placement. Participants listen to two-sentence discourses containing in ambiguous pronoun. The second sentence varies between a neutral condition and a focus condition.

### Participants

Seventy-five English-speaking participants were recruited from Amazon Mechanical Turk, location restricted to the United States. Data was eliminated from seven bilinguals and three participants who either did not complete or did not understand the task. Participants were paid between $1.25 and $2.00.

### Materials

Target stimuli consisted of 16 recorded passages, as in (21). The first sentence mentioned two referents in a situation described with an NP2-biased IC verb. The two referents were of the same gender, counterbalanced between male and female names. The second sentence started with an ambiguous pronoun followed by a continuation that was intended to be plausible under either interpretation of the pronoun.

(21) Charles congratulated Simon. He had criticized Stephanie.

The passage varied between a neutral condition and a focus condition. The focus condition was uttered with the intention of conveying that the two sentences both provided answers to a question about what the first referent had done. For this manipulation to work, the preferred pronoun interpretation with neutral intonation must be to the non-subject. That is precisely why the class of NP2-biased IC verbs provides an ideal test case.

The target stimuli were interleaved with 16 fillers that were produced with either neutral or focus-marked intonation.

### Procedure

Participants were asked to listen to a sentence and answer a question about the speaker's intended meaning in the provided text box. As in Experiments 1 and 2, the text showing the question was visible on the screen during and after playback of the recorded sentence, and participants could replay the sentence as many times as they wished.

For the Experiment 3 target items, the question asked who did the action described in the second sentence: For example, the question for the recording of (21) was (22).

(22) Who criticized Stephanie?

The task took roughly 15 min.

### Results

Responses to target items were coded as subject [e.g., an answer of “Charles” to (22)], object (“Simon”), or unknown (e.g., “the teacher”). The responses to filler trials were also coded and used to determine which participants to exclude from analysis. Odd responses were taken to indicate that a participant might not have been paying sufficient attention or might not have been able to hear the audio sufficiently well. For example, a participant presented with a filler like “Paul leaned across the table toward Stacie. He then asked her to marry him.” who answered the question of “Who proposed?” with “Stacie” had that answer coded as an outlier (misinterpreting “he” as “she”). Likewise, a participant presented with a filler like “Vicki is attracted to Dennis. He is repulsed by her.” who answered the question “Who is repulsed?” with “Becky” had that answer coded as an outlier (mishearing Vicki as Becky). After eliminating the 14 participants with 2 or more outlier answers on filler trials, data from 50 participants remained for the analysis. Responses categorized as unknown (0.5% of target trials) were also removed.

**Figure 3:**
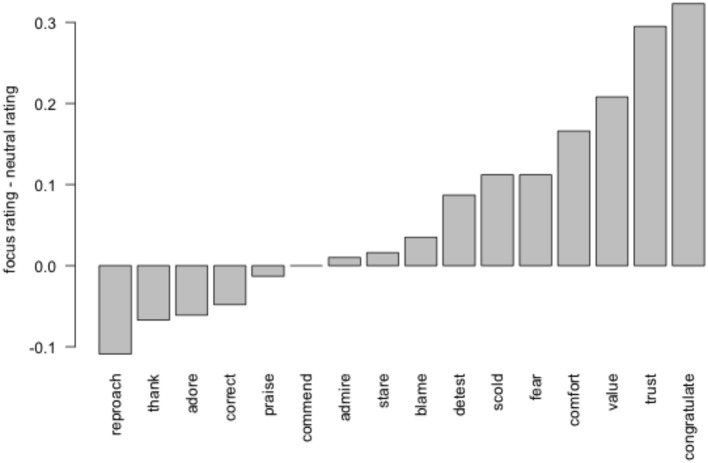
**Subject coreference in Experiment 3, by item**.

In keeping with previous studies on NP2-biased IC verbs, participants favored the object as the referent of the pronoun (62% object coreference overall). As predicted, however, pronouns were interpreted to refer to the subject more often in the focus condition (mean = 41%) than the neutral condition (mean = 35%). A mixed-effect logistic regression showed a main effect of condition (β = −0.47, *p* < 0.05, based on the Wald Z statistic; Agresti, 2002). Figure 3 shows the difference between ratings in the focus placement and neutral conditions, broken down by item.

## Discussion

The results of our experiments broadly support our hypothesis that focus-driven pragmatic effects would be observable in all three domains of interest. In the case of scalar implicature, we see a general tendency for focus marking of the weak scalar to promote interpretations involving the implicature. In the case of presupposition, focus marking within the presupposed material—under the scope of negation—tends to promote interpretations in which the presupposition fails to project to the discourse level. In the case of subject pronoun disambiguation, focus marking on the sentential object tends to promote parallel interpretations of the pronoun.

As explored earlier, all these patterns are explicable in terms of QUD effects. This relies crucially upon the assumption that the intonation employed as an indicator of focus structure in the materials used is actually used by hearers as an indication of which QUD is currently in play. In principle, this appears to be a reasonable assumption: Most and Saltz (1979) documented experimentally that hearers were able to infer the questions to which differently-intoned sentences were answers. It is important to reiterate that our materials were not constructed in such a way as to control their prosodic properties: the sentences were merely read by a native speaker who was trying to convey an intended meaning as opposed to trying to realize a specific contour. Consequently, we are not licensed to draw precise conclusions about the relationships between prosody, focus, and QUD. We can, however, conclude that a purely intonational manipulation that targets a particular constituent can have pragmatic effects on the hearers which are predictable under a QUD-based account.

The observed effects are in keeping with existing work showing that focus factors matter to interpretation by activating alternatives. Such effects have been demonstrated for the inclusive/exclusive interpretation of “or” (Chevallier et al., 2008, 2010) and for the exhaustivity inferences of “only” and the additive presuppositions “also” (Gotzner and Spalek, 2014; see also Tomlinson and Bott, 2013). With respect to scalar implicatures, our work complements ongoing research on the role of prosody in such contexts: results reported by de Marneffe and Tonhauser (2015) demonstrate that a specific prosodic contour can increase the availability of scalar implicatures compared to a neutral intonation contour, although the effect that they document is evident when the discourse context is also provided. As de Marneffe and Tonhauser note, this suggests that the prosodic influence on implicature is a more complex matter than simply whether or not the weak scalar receives a pitch accent, as this was the case for the all the conditions in their experiment. This in turn suggests an important role for research on scalar implicatures using auditory stimuli, as the kind of intonation contour inferred by a participant reading written stimuli cannot always be determined with confidence.

Regarding the mechanisms by which hearers generate pragmatic enrichments, there are several possibilities that are compatible with the kinds of pragmatic enrichments that we observe. For the much-discussed case of scalar implicature, possible strategies include interpreting the weak scalar as semantically (or typically—see Geurts and van Tiel, 2013) excluding the possibility that the strong scalar holds, or inserting a tacit exhaustivity operator over the scalar when parsing the sentence (Chierchia et al., 2012). Compared to these options, the QUD-based account appears computationally more laborious, and is more in keeping with the traditional Gricean approach to quantity implicatures. However, explaining the effect of focus within these other approaches is perhaps not so straightforward: we would have to construe it as inducing either a particular interpretative preference or a particular parsing preference at the weak scalar term itself. Consequently, it seems plausible to treat the patterns observed in this experiment as supportive of the QUD-based model.

There are similarly several different routes by which a given presupposition can project to the discourse level, as discussed earlier in this paper. On one account, the hearer adds the presupposition to her discourse model immediately upon encountering the trigger, even if it occurs under the scope of negation; but this proposal runs into difficulty in cases of local accommodation (i.e., where the presupposition turns out not to be intended by the speaker). Another possibility is that the hearer considers whether the QUD that they infer on the basis of the utterance carries the presupposition, and if so, this enables them to project the presupposition from under the scope of negation. Still another possibility arises for a particular class of utterances with presupposition triggers, as in the case of (23), contrasted here with (24).

(23) Mary doesn't regret that the Tories won the election.
(24) Mary doesn't regret arguing with her boss.

If “Mary” is stressed in (24), a possible interpretation is that it is someone other than Mary who regrets arguing with their boss. Under this interpretation, (24) does not convey that Mary argued with her boss: indeed, it does not convey that anyone argued with Mary's boss, although it does convey that someone argued with his or her own boss (which may or may not be the same individual as Mary's boss). By contrast, applying the same reasoning to (23), the utterance may convey that someone other than Mary regrets that the Tories won the election, and this in turn requires it to be the case that the Tories won the election. In effect, it appears possible that the utterance with focal stress triggers some kind of ad hoc implicature which in turn introduces a presupposition into the hearer's discourse model.^4^ In the examples tested in this paper, this possibility does not arise, but the prediction would be that focal stress on “Mary” should make no difference to the projection of the presupposition in (23).

## Conclusion

The three experiments reported here test how a manipulation of focus placement can influence three phenomena that involve pragmatic enrichment, all of which are sensitive to the QUD evoked by the context. Unlike previous work that has explicitly manipulated the previous context, here it is the focus placement itself that informs the listener about the possible QUD to which the current sentence may be an answer. The repercussions of this QUD manipulation can be seen in three different types of pragmatic enrichment: scalar inference, the projection of presupposition from under negation, and the identification of a referent for an ambiguous pronoun. In each case, focus placement signals what QUD is likely and that QUD in turn determines the relevance of a particular proposition for the interpretation of the target sentence. For scalar implicature, highlighting the relevance of an unstated alternative that is informationally stronger is found to heighten the availability of the implicature. For presupposition, highlighting the relevance of an alternative which does not itself carry the presupposition reduces projection. For coreference, highlighting the relevance of a particular alternative favors the inference of a parallel coherence relation between two adjacent sentences, thereby disfavoring coreference with the referent picked out by causal reasoning. Together, these experiments show that a single manipulation can influence a varied set of phenomena. Our findings suggest that the study of context can and should move beyond ad hoc explanations for specific readings and toward the identification of cues that alter context in systematic ways. Of course not all context-driven effects depend on focus placement, but the results reported here offer a first step toward a necessary inventory of targeted contextual manipulations that guide listeners' interpretations.

### Conflict of interest statement

The authors declare that the research was conducted in the absence of any commercial or financial relationships that could be construed as a potential conflict of interest.

## Footnotes

^1^Of course, the speaker's unwillingness to make a stronger statement may have the effect of signaling their lack of certain knowledge about the truth or falsity of the stronger statement.

^2^The recordings of the target stimuli for all three experiments are available here: http://dx.doi.org/10.7488/ds/315.

^3^One of our reviewers points out that the direction of the scale may not be obvious for the items “delayed” (“not on time” vs. “not canceled”) and “smoldering” (“not alight” vs. “not out”). The participants in the experiment were asked how likely it was that the “train was not canceled” and “the fire was not out,” respectively. Removing those two items does not affect the overall analysis: The main effect of intonation is still significant. Note that “smoldering,” which is arguably the harder one to identify its pragmatically strengthened meaning, is shown in Figure 1 to be the worst performing item, so it is possible that asking the question differently (“how likely was it that the fire was not alight?”) might have yielded different responses.

^4^This inference requires the speaker to be knowledgeable; the assumption would be that such a speaker, knowing that no-one (of interest) argued with their boss [in (B)] or that the Tories didn't win the election [in (A)], would say so.
