# Supplementary material for: Evoking Context with Contrastive Stress: Effects on Pragmatic Enrichment
Source: Front Psychol. 2015 Nov 26;6:1779. doi: 10.3389/fpsyg.2015.01779 (PMC4659916; doi:10.3389/fpsyg.2015.01779)
Supplement: Supplementary file 1 [file DataSheet1.DOCX]

**Appendix A**

*Implicature stimuli*

The software we need is cheap. (How likely is it that the software is not free?)

The species that the naturalist studies is rare. (How likely is it that the species is not extinct?)

The coffee we were served is warm. (How likely is it that the coffee is not hot?)

The medicine that the doctor recommends is scarce. (How likely is it that the medicine is not unavailable?)

The essay that the student submitted is good. (How likely is it that the essay is not great?)

The movie that we watched last night is unsettling. (How likely is it that the movie is not terrifying?)

The project that the council planned is underway. (How likely is it that the project is not finished?)

The city we visited is old. (How likely is it that the city is not ancient?)

The museum of fine art is big. (How likely is it that the museum is not immense?)

The view from the hotel window is pretty. (How likely is it that the view is not gorgeous?)

The consultant advising the company is intelligent. (How likely is it that the consultant is not brilliant?)

The apartment we looked at is small. (How likely is it that the apartment is not tiny?)

The team's performance is adequate. (How likely is it that the team's performance is not amazing?)

The piece of music the pianist played is difficult. (How likely is it that the piece is not impossible?)

The soil in the flowerbed is damp. (How likely is it that the soil is not soaking?)

The train is delayed. (How likely is it that the train is not canceled?)

The bonfire is smouldering. (How likely is it that the bonfire is not out?)

The use of Linux is permitted. (How likely is it that the use of Linux is not required?)

The work done by the decorators is satisfactory. (How likely is it that the work is not excellent?)

The dog owned by our neighbours is overweight. (How likely is it that the dog is not obese?)

*Presupposition stimuli*

Bill doesn’t regret arguing with his boss.

Mary doesn’t know that the tax auditor is watching her company.

Raj isn’t aware that the electrician is coming Tuesday.

Kesha isn’t happy that the landlord is remodelling her kitchen.

Craig isn’t relieved that the police cancelled his parking fine.

Antonia isn’t sorry that her jewels are in the safe.

Steven didn’t manage to pass his exams.

Ella didn’t forget her boyfriend’s birthday.

Terence didn’t avoid seeing his ex in the mall.

Rachel didn’t stop smoking cigarettes.

Kevin didn’t finish his degree at Harvard.

Poppy didn’t return her necklace.

Aaron didn’t leave the performance of the Nutcracker.

Julie didn’t arrive in the country last year.

Richard didn’t get drunk again at the office party.

Helen didn’t return to her job at Chrysler.

Luther didn’t go back to the brokerage firm.

Tanya didn’t rewrite her paper on surrealism.

George wasn’t restored to his position as vicepresident.

Yolanda didn’t repeat her criticism of the government.

*Coreference stimuli*

Mary scolded Sue. She had talked to Justin.

Debbie comforted Nicole. She had placated Jim.

Sally corrected Amy. She had tattled on Jeff.

Jessica thanked Ashley. She had credited Rick.

Jennifer commended Debbie. She had stood up to Arthur.

Amanda fears Beth. She spends time with Kevin.

Kate admires Elizabeth. She hates Robert.

Melissa adores Susan. She puts up with Todd.

Josh trusted David. He had accused Lucy.

Ryan reproached George. He had complimented Sarah.

Charles congratulated Simon. He had criticized Stephanie.

Frank blamed Greg. He had favored Holly.

Adam praised Bill. He had confronted Theresa.

Alan stared at Carl. He had ignored Julia.

John detests Bob. He likes Tracy.

Luis values Rob. He appreciates Courtney.
